# Estimation of umbilical cord blood leptin and insulin based on anthropometric data by means of artificial neural network approach: identifying key maternal and neonatal factors

**DOI:** 10.1186/s12884-016-0967-z

**Published:** 2016-07-21

**Authors:** José Guzmán-Bárcenas, José Alfredo Hernández, Joel Arias-Martínez, Héctor Baptista-González, Guillermo Ceballos-Reyes, Claudine Irles

**Affiliations:** Department of Physiology and Cellular Development, Instituto Nacional de Perinatología Isidro Espinoza de los Reyes (INPerIER), Montes Urales 800, Lomas de Virreyes, Mexico city, C.P. 11000 Mexico; Centro de Investigación en Ingeniería y Ciencias Aplicadas (CIICAp)-Universidad Autónoma del Estado de Morelos (UAEM), Cuernavaca, Morelos Mexico; Departmento de Ciencias de la Salud-Universidad de Sonora, Campus Cajeme, Sonora Mexico; Laboratorio Multidisciplinario y Sección de Estudios de Posgrado e Investigación, Escuela Superior de Medicina, Instituto Politécnico Nacional, Mexico City, Mexico

**Keywords:** Mathematical model, Leptin, Insulin, Neonate, Artificial neural network, Umbilical cord blood, Gestational diabetes, Maternal obesity

## Abstract

**Background:**

Leptin and insulin levels are key factors regulating fetal and neonatal energy homeostasis, development and growth. Both biomarkers are used as predictors of weight gain and obesity during infancy. There are currently no prediction algorithms for cord blood (UCB) hormone levels using Artificial Neural Networks (ANN) that have been directly trained with anthropometric maternal and neonatal data, from neonates exposed to distinct metabolic environments during pregnancy (obese with or without gestational diabetes mellitus or lean women). The aims were: 1) to develop ANN models that simulate leptin and insulin concentrations in UCB based on maternal and neonatal data (ANN perinatal model) or from only maternal data during early gestation (ANN prenatal model); 2) To evaluate the biological relevance of each parameter (maternal and neonatal anthropometric variables).

**Methods:**

We collected maternal and neonatal anthropometric data (*n* = 49) in normoglycemic healthy lean, obese or obese with gestational diabetes mellitus women, as well as determined UCB leptin and insulin concentrations by ELISA. The ANN perinatal model consisted of an input layer of 12 variables (maternal and neonatal anthropometric and biochemical data from early gestation and at term) while the ANN prenatal model used only 6 variables (maternal anthropometric from early gestation) in the input layer. For both networks, the output layer contained 1 variable to UCB leptin or to UCB insulin concentration.

**Results:**

The best architectures for the ANN perinatal models estimating leptin and insulin were 12-5-1 while for the ANN prenatal models, 6-5-1 and 6-4-1 were found for leptin and insulin, respectively. ANN models presented an excellent agreement between experimental and simulated values. Interestingly, the use of only prenatal maternal anthropometric data was sufficient to estimate UCB leptin and insulin values. Maternal BMI, weight and age as well as neonatal birth were the most influential parameters for leptin while maternal morbidity was the most significant factor for insulin prediction.

**Conclusions:**

Low error percentage and short computing time makes these ANN models interesting in a translational research setting, to be applied for the prediction of neonatal leptin and insulin values from maternal anthropometric data, and possibly the on-line estimation during pregnancy.

**Electronic supplementary material:**

The online version of this article (doi:10.1186/s12884-016-0967-z) contains supplementary material, which is available to authorized users.

## Background

The impact of maternal obesity during pregnancy (maternal overweight/obesity with or without gestational diabetes mellitus) and its association with an increased risk of obesity, as well as re-programming cardiovascular risk, body composition and cardiometabolic health in infancy and early adulthood, has been shown in humans and animal models [1–7]. It has been demonstrated that human obesity in children and adults is associated with elevated serum levels of an adipokine, the hormone leptin, reflecting the amount of energy stored in adipose tissue [8, 9]. Leptin was identified as the product of the obesity (*ob*) gene [10], which is secreted into the circulation by large adipocytes, and has been shown to cross the blood–brain barrier and bind to specific receptors in the hypothalamus to alter the expression of several neuropeptides that regulate neuroendocrine function, energy intake and expenditure leading to a decrease in appetite, reduction of body fat and body weight [11, 12]. The pancreatic hormone insulin also acts in the brain as a negative feedback signal for adipocity. It is also an essential regulator of growth, increasing fat deposition [13] resulting in a greater potential for leptin synthesis by stimulating adipocyte *ob* gene transcription [14]. As well, leptin also modulates (increases) insulin secretion by pancreatic β cells [15]. Therefore, leptin and insulin control glucose metabolism, acting at the peripheral and central level [16]. During pregnancy, leptin levels regulate fetal development and growth [17, 18] and positively correlate in umbilical cord blood (UCB) with neonatal body weight and fat mass [9]. It has been demonstrated that UCB leptin concentration correlates with insulin levels and anthropometric data (birth weight) only in large for gestational age neonates, but they do not correlate with maternal levels [19–21]. Both leptin and insulin biomarkers are used as predictors of weight gain and obesity during infancy. Indeed, several studies have shown that lower cord blood leptin levels predict an increased weight and length gain, “catch-up” growth, as well as a higher BMI in infancy (2–3 years) [22–24]. Yet, in the first months, decreased cord leptin levels together with gestational diabetes mellitus are related to a slower weight gain [25]. For insulin, an inversely relationship was found for weight gain during infancy [26]. Therefore, the prediction of cord blood hormone levels based on anthropometric maternal and neonatal data using mathematical models that take into account the high complexity of this system may be of considerable usefulness. Therefore, Artificial Neural Networks (ANNs) will be used as a system biology approach to simulate cord blood hormone levels.

ANNs [27] have been extensively used for the optimization and modeling of processes, as they are able to represent the non-linear dynamic interaction of complex relationships without any assumptions of the underlying mechanisms [28]. ANNs learn and test the solution of the problem from a data set [29] and provide an interpolation for new data. For the science of medicine, the application of neural networks keeps on expanding [30–32], and now represents a set of methods that have been useful for solving pediatric problems [33, 34], identifying key factors such as in fetal growth [35, 36] and diagnosing neonatal diseases [37].

ANN has the ability to predict data such as measuring biochemical parameters in UCB samples which may be difficult to obtain otherwise. The objectives of the study were therefore: 1) to obtain ANN models (feed-forward) for the prediction of leptin and insulin values in UCB from neonates exposed to distinct metabolic environments during pregnancy (defined as obesity with or without gestational diabetes mellitus or lean women), based on anthropometric maternal and neonatal characteristics (ANN perinatal model) or from only maternal data during early gestation (ANN prenatal model) 2), to examine which parameters, among those analyzed from the mother and neonate, have the most influence on neonatal leptin and insulin values by applying a sensitivity analysis. Essentially, ANN will learn from a database (maternal and neonatal clinical data as well as biochemical experimental data) from a specified problem (maternal metabolic environment) with a known solution (UCB leptin and insulin experimental values for training the model) and then the network, will recreate the system of an inherent complex set of data (testing the model).

## Methods

### Study subjects

This study was approved by both the Ethics and Research Committees of the Instituto Nacional de Perinatologia *“Isidro Espinosa de los Reyes”*. Venous umbilical cord blood samples were collected from 49 cesarean deliveries. Samples were centrifuged (for 15 min, at 3500 rpm, room temperature), serum was aliquoted and stored at −70 °C until assayed. Hemolyzed or lipemic samples were discarded. Mothers (*N* = 49) were: lean normoglycemic (initial Body Mass Index, BMI, of 24.3 ± 0.4 kg/m^2^, *n* = 11), obese (initial BMI of 30.9 ± 0.9 kg/m^2^, *n* = 23) and obese with gestational diabetes mellitus (initial BMI of 31.3 ± 0.7 kg/m^2^, *n* = 15). We collected the following information: maternal morbidity (MM), gestational age at delivery (GE), initial and final maternal weight (MWi and MWf), initial and final BMI (MBMi and MBMf), maternal height (MH), maternal age (MA), parity (P), neonatal gender (NG), neonatal birth weight (NW), neonatal body length (NH), neonatal head circumference (NHC), neonatal BMI (NBMI) and 5-min APGAR score. The age range of the participants was 16–43 years. Exclusion criteria were genetic syndromes, chromosomal abnormalities, gross placental abnormalities, infections and substance abuse. The main clinical data are reported in Table 1.

**Table 1:** Maternal and neonatal clinical data

		Mother	
Parameters	Healthy	Obese	Diabetic
*N = 49*	11	23	15
Maternal age (years)	25.1 (±3.3)	30.3 (±1.1)	35 (±1.2)
Maternal initial weight (kg)	58.3 (±2.1)	74.2 (±2.5)	77.3 (±2.7)
Maternal final weight (kg)	67.9 (±2.6)	85.6 (±3.2)	89.4 (±3.9)
Maternal height (cm)	156.4 (±2.2)	157.1 (±1.3)	157.3 (±1)
Gestational age at delivery, (weeks)	38.8 (±0.2)	38 (±0.4)	39 (±0.3)
Initial Maternal BMI (kg/m^2^)	24.3 (±0.4)	30.7 (±0.8)	31.3 (±0.8)
Final Maternal BMI (kg/m^2^)	27.7 (±0.5)	34.7 (±1.2)	36 (±1.4)
Parity	1.7 (±0.3)	2.7 (±0.2)	2.6 (±0.3)
Males/Females	5M/6F	7M/8F	11M/11F
Neonatal birth weight (kg)	2.87 (±0.14)	2.88 (±0.09)	3.22 (±0.09)
Neonatal birth body length (cm)	47.9 (±0.6)	47.9 (±0.4)	48.3 (±0.7)
Neonatal head circumference (cm)	34.4 (±0.3)	33.8 (±0.2)	34.7 (±0.3)
Neonatal BMI	12.45 (±0.4)	12.52 (±0.3)	13.88 (±0.4)
5-min APGAR score	9	8.9 (±0.07)	9 (±0.06)

All values are depicted as Mean +/- SEM

### Leptin and Insulin determination by ELISA

Leptin and insulin from umbilical cord blood samples were assayed using commercially available ELISA kits (GenWay, San Diego, CA). The sensitivity of the leptin assay was 0.1 ng/ml; intra-assay and inter-assay coefficients of variations were 4.2 and 6.7 %, respectively. The sensitivity of the insulin assay was 0.3 μU/ml; intra-assay and inter-assay coefficients of variations were 6.3 and 8.5 %, respectively.

### Database

We compared neonates and their mothers with distinct metabolic environments during pregnancy (defined as obese, obese with gestational diabetes mellitus or lean women). Fourteen variables were selected from the entire database for 49 subjects. For the ANN models, anthropometric, maternal morbidity (obese, obese with gestational diabetes mellitus or lean women) and biochemical data (umbilical cord blood leptin or insulin) were administered.

### ANN learning and testing

**Fig. 1:**
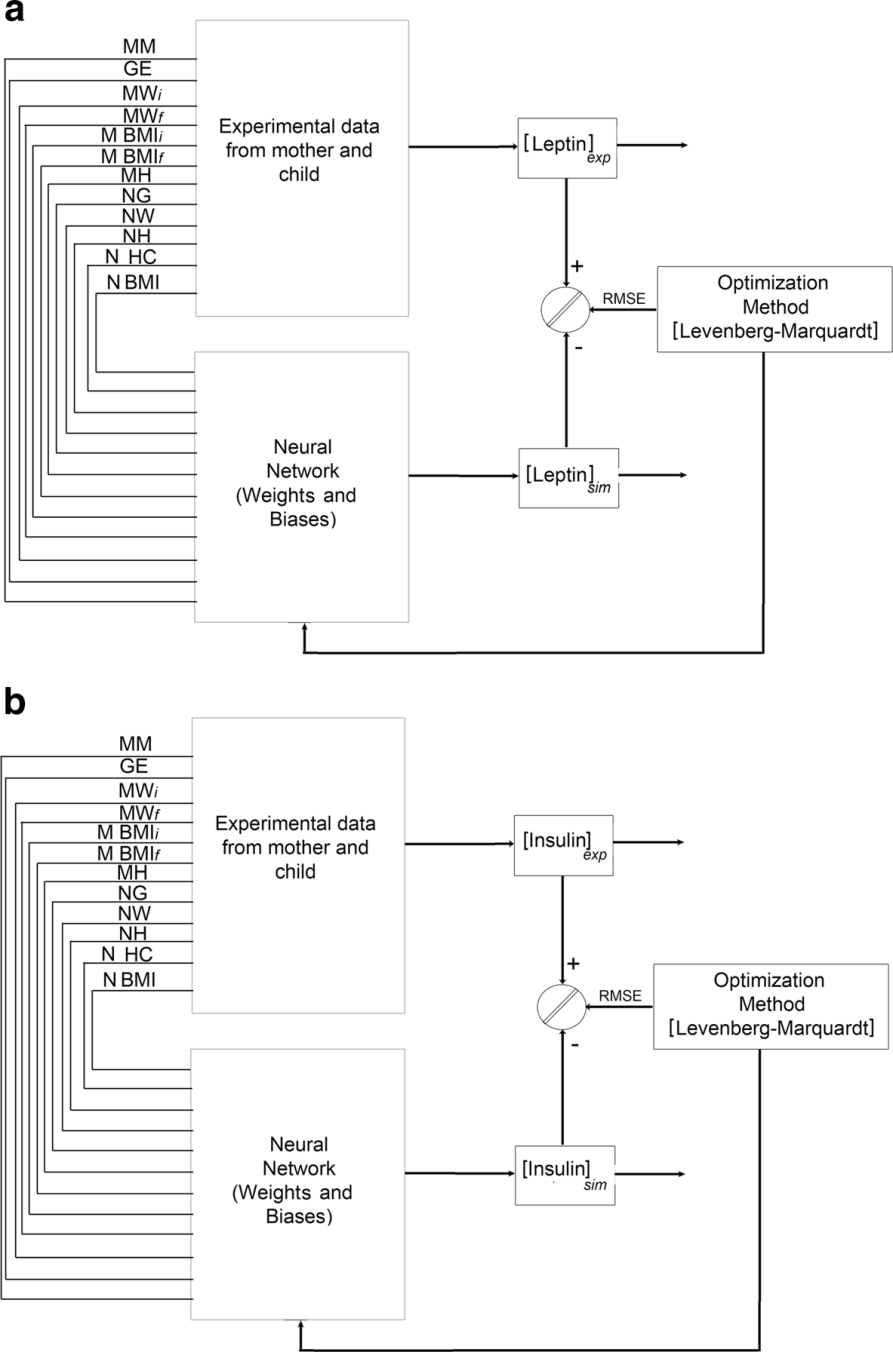
Recurrent network architecture of the ANN perinatal model and the procedure used for learning neural network for the simulation of leptin (**a**) and insulin (**b**) concentration in umbilical cord blood (UCB) samples

Back-propagation (BP) algorithm multiple-layer perceptron (MLP) architecture was trained and tested by the input layer, the hidden layer and the output layer (see Fig. 1). We applied the Log-sigmoid (LOGSIG) and hyperbolic tangential (TANSIG) transfer functions in the hidden layer. Both transfer functions were acceptable however; the hyperbolic tangential performance was slightly superior. According to [38], TANSIG transfer function has a better performance which is in agreement with our result. In the output layer, only the linear transfer function (PURELIN) was employed because the output layer is not normalized. In order to obtain the optimum model, we began in the hidden layer with one neuron until the Root Mean Square Error (*RMSE*) did not change and the statistical test (slope and intercept [39]) was approved, as well as we avoided over-fitting (for a detailed explanation see Additional file 1) and [40–43]. All calculations were carried out with Matlab mathematical software (Natick, MS, USA) with the Neural Network Toolbox for Matlab [40].

The input layer for the ANN perinatal models consisted of 12 maternal and neonatal variables and the output layer contained one variable for umbilical cord blood leptin or umbilical cord blood insulin concentrations. The variables were: maternal morbidity (MM), gestational age at delivery (GE), initial and final maternal weight (MWi and MWf), initial and final maternal BMI (MBMi and MBMf), maternal height (MH), neonatal gender (NG), neonatal birth weight (NW), neonatal body length (NH), neonatal head circumference (NHC) and neonatal BMI (NBMI) (Table 2). The input layer for the ANN prenatal models (early gestation) contained 6 maternal variables (maternal morbidity (MM), initial maternal weight (MWi), initial maternal BMI (MBMi), maternal height (MH), maternal age (MA) and parity, P) and the output layer had one variable for umbilical cord blood leptin or umbilical cord blood insulin concentrations (Table 2).

**Table 2:** List of experimental variables (clinical and biochemical data) analyzed using ANN to obtain umbilical cord blood leptin and insulin values: input and output range conditions studied

Input Variables (*n* = 49)	Range	Output variables	Range
Maternal Morbidity, MM	Healthy, Obese or diabetic	Umbilical cord blood leptin, (ng/ml)	0.17–27 (mean 5.1)
Maternal initial weight, MWi (kg)	49–96 (mean 72)		
Maternal final weight, MWf (kg)	55–117 (mean 83)	Umbilical cord blood insulin, (μU/ml)	0.7–12 (mean 1.9)
Maternal height, MH (cm)	149–173 (mean 157)		
Maternal initial BMI, MBMi (kg/m^2^)	22–40 (mean 29.5)		
Maternal final BMI, MBMf (kg/m^2^)	24–42 (mean 33.5)		
Gestational age at delivery, GE (weeks) Neonatal gender, NG	37–41 (mean 39)		
Neonatal birth weight, NW (kg)	2.01–4.19 (mean 2.98)		
Neonatal birth body length, NH (cm)	45–54 (mean 48)		
Neonatal head circumference, NHC (cm)	32–37 (mean 34)		
Neonatal BMI, NBMI	10–15 (mean 13)		
Parity, P	1–5 (mean 2.3)		
Maternal age, MA (years)	16–43 (mean 30)		

In the learning, to change the weights and biases, we applied the Levenberg-Marquardt (LM) algorithm as the learning (training) algorithm allowing to obtain a smaller *RMSE* [40, 44, 45] (for a detailed explanation see Additional file 1). The *RMSE* was calculated from the experimental values and network predictions (see Fig. 1a and b).

The experimental database (*n* = 49) was used to feed the ANN structure. This database (*x*_*i*_) was randomly divided into: learning (79 %) and validation (21 %). The database was then normalized in the range of 0.1 to 0.9 [46] for the input variables and the output variable was not normalized.

So, the entire input database was scaled to a new value *x*_*i*_ as follows:

1 $$ {x}_i=0.8\;\left(\frac{X_i-{X}_{\min }}{X_{\max }-{X}_{\min }}\right)+0.1 $$

### Statistical test

In order to confirm the best performance of the ANN predictions, a linear regression was carried out to obtain the slope and intercept from the ANN simulations versus the experimental database (learning and validation database), after which we applied a statistical test (slope and intercept, [39, 47]). This last consists in demonstrating that the obtained upper and lower intervals of the slope must be one and the upper and lower intervals of the intercept must be zero, with a 99.8 % confidence level according to the Student t-test.

## Results

The main umbilical cord blood leptin and insulin experimental values are reported in Table 3 and are in agreement with the study by [48] (as well as [49–51]). Briefly, obesity exposed neonates had higher UCB leptin levels compared to not exposed neonates and a trend for increased levels in gestational diabetes exposed neonates. Gestational diabetes exposed neonates had the highest values for umbilical cord blood insulin levels than not exposed or obesity exposed neonates.

**Table 3:** Umbilical cord blood hormone concentrations

		Mother	
Parameters	Healthy	Obese	Diabetic
*N = 49*	11	23	15
Leptin (ng/ml)	3.5 (±1)	6.7 (±1.5)	4.9 (±0.4)
Insulin (μU/ml)	1.3 (±0.4)	1.06 (±0.08)	3.6 (±1)

### Proposed ANN perinatal model

**Fig. 2:**
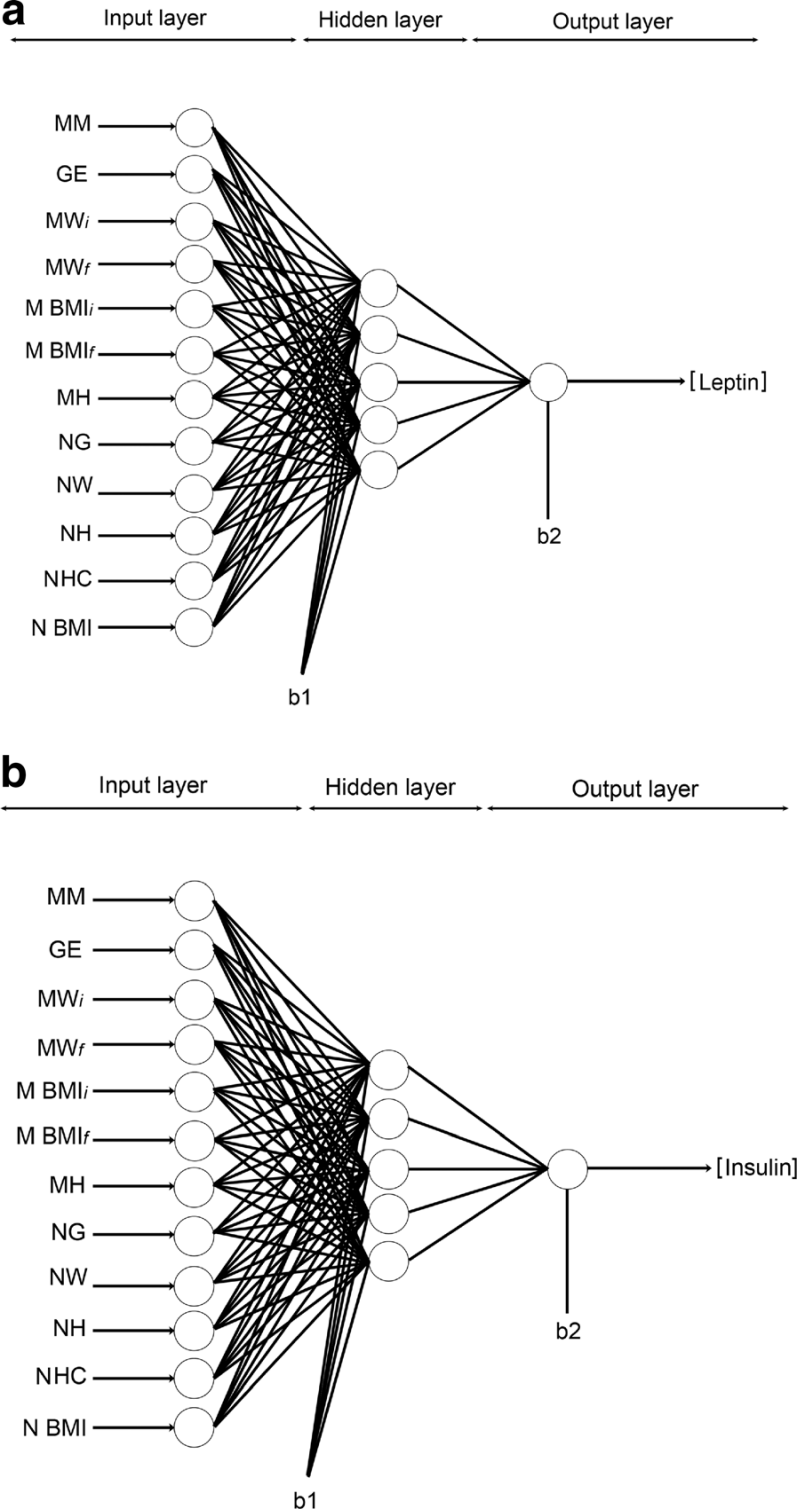
The neural network computational ANN perinatal model for UCB leptin (**a**) and insulin (**b**) concentration estimation. The proposed model involved 12 input variables, 5 neurons on hidden layer and 1 output variable

The input variables for the ANN perinatal models were 12 (maternal and neonatal) and were presented to the general network, in which the final UCB hormone level prediction corresponded to the output unit: 1 output variable for UCB leptin or insulin concentration. Figure 2 shows the general scheme of such neural network architecture for the prediction of UCB leptin (Fig. 2a) and insulin (Fig. 2b) values from perinatal parameters (as depicted in Fig. 1a and b). 20,000 runs with 100 iterations were applied in each neuron from 1 to 5 neurons in the hidden layer and the final topology was obtained for leptin and insulin predictions. As a result, the best network architecture performance was 12-5-1 for both models: leptin and insulin (Equations [6–11] and the weights and biases are reported in Additional file 1: Tables S1 and S2).

### Validation of the ANN perinatal model

**Fig. 3:**
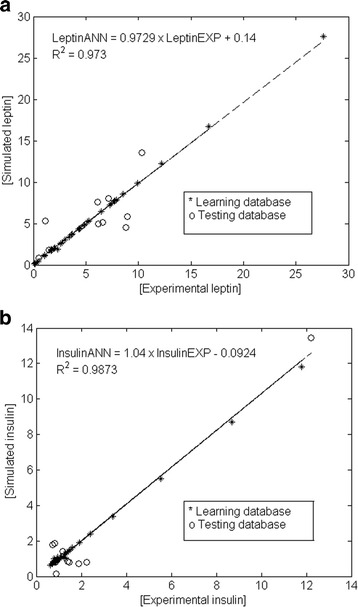
The scatter plot of perinatal experimental (open circles) vs. ANN-predicted values (dark cross) for average UCB leptin (**a**) and insulin (**b**). Experimental (leptin_EXP_ and insulin_EXP_) and simulated (leptin_ANN_ and insulin_ANN_) data. Dashed line indicates the fitted simple regression line on scattered points

Figure 3 depicts the comparison of the experimentally measured (_EXP_) and the predicted (_ANN_) UCB leptin (Fig. 3a) and insulin (Fig. 3b) values for the testing database describing the behavior of the ANN perinatal model using all data available (inputs).

The comparison of (_EXP_) and (_ANN_) data through a linear regression model,

(*Leptin*_*ANN*_ = *a* + *b Leptin*_*EXP*_) and (*Insulin*_*ANN*_ = *a* + *b Insulin*_*EXP*_), showed regression coefficients of *R*^2^ > 0.973 and *R*^2^ > 0.9873 for leptin and insulin, respectively. Upper and lower values of the statistical test (Table 4) indicate that the slope included one and the intercept contained zero, with a 99.8 % confidence level for both UCB determinations [39, 47]. These results demonstrated a good correlation between ANN predictions and experimental values.

**Table 4:** Intercept (a) and slope (b) statistical test to leptin and insulin in the ANN perinatal model

Leptin		Insulin	
a_lower_	a_upper_	a_lower_	a_upper_
_−0.6321_	_0.9118_	_−0.3411_	_0.1564_
b_lower_	b_upper_	b_lower_	b_upper_
_0.8624_	_1.0833_	_0.9564_	_1.1156_

### Sensitivity analysis of the ANN perinatal model

**Fig. 4:**
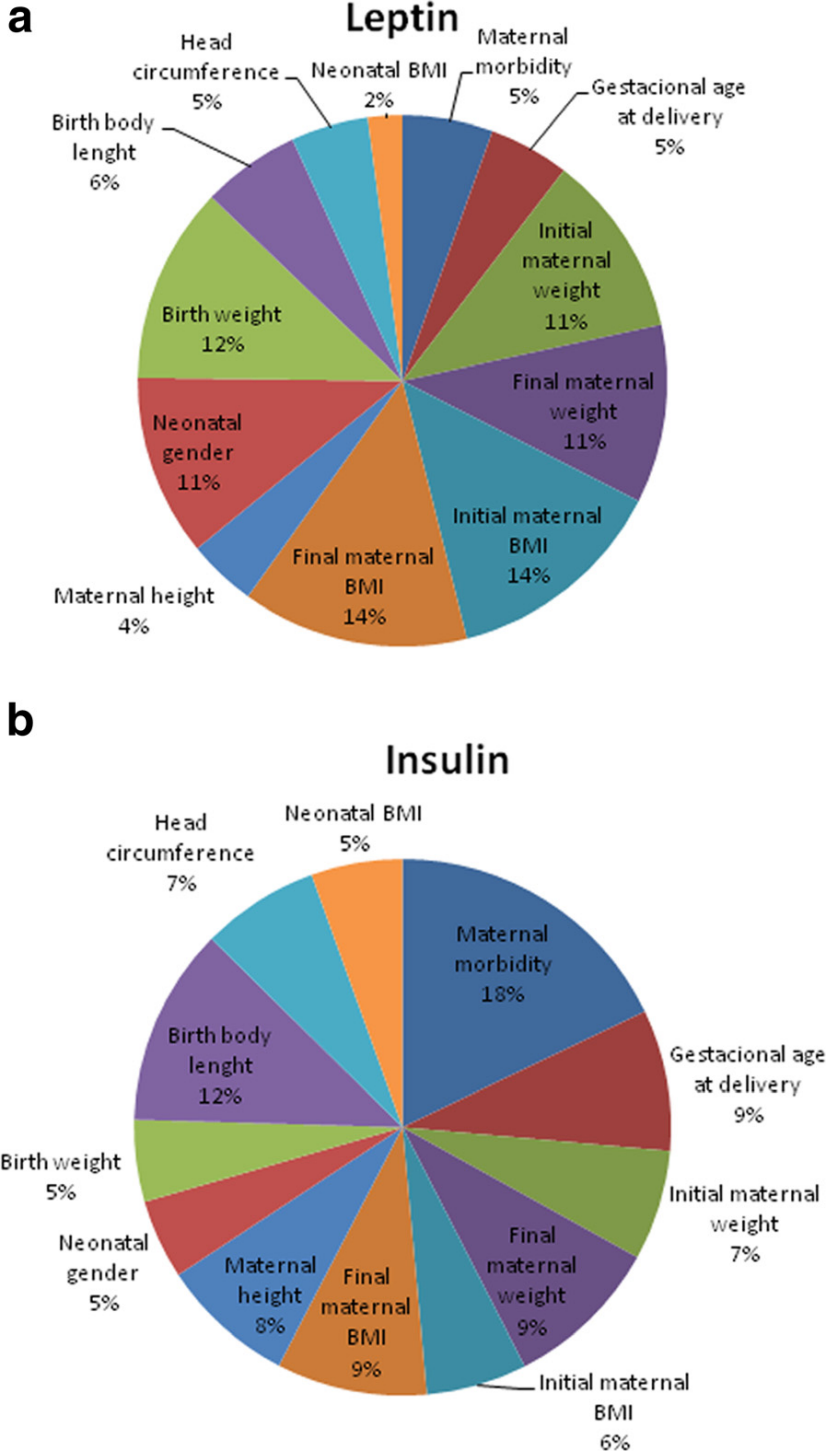
Percentage for the global sensitivity analysis of the 12 input variables in the ANN perinatal model for UCB leptin (**a**) and Insulin (**b**) values

We used an evaluation process based on the neural network weight matrix and the Garson equation [52, 53] to obtain the qualitative significance of the input variables on the predicted UCB leptin and insulin values (for a detailed explanation of Equation [19], see Additional file 1). Figure 4 depicts the relative importance of the calculated input variables showing that all variables had a strong effect on leptin (Fig. 4a) and insulin (Fig. 4b) neonatal values. In addition, the sensitivity analysis showed that maternal BMI (28 %, initial and final BMI), neonatal birth weight (12 %) and maternal weight (11 %) were the most influential factors controlling umbilical cord blood leptin concentration, in contrast with maternal morbidity (healthy, obese or controlled gestational diabetes, 5 %) and neonatal BMI (2 %) that were the less important factors for estimating UCB leptin levels (Fig. 4a).

For UCB insulin concentration, maternal morbidity (healthy, obese or controlled gestational diabetes) appears to be the critical parameter with a relative importance of 18 %, followed by neonatal body length at delivery (12 %), maternal BMI (6–9 %) and less importantly neonatal BMI (5 %) and birth weight (5 %) (Fig. 4b).

Altogether, these results showed that the ANN perinatal models succeeded in predicting the experimental results of UCB leptin and insulin concentration from anthropometric maternal and neonatal values, as well as revealed a good agreement between the experimental data and the predicted values. However, this ANN perinatal models required gestation and at term information in order to predict UCB leptin and insulin concentrations. Therefore, a second ANN model was applied to predict UCB levels using only inputs from early gestation of the same database.

### Proposed ANN prenatal model

**Fig. 5:**
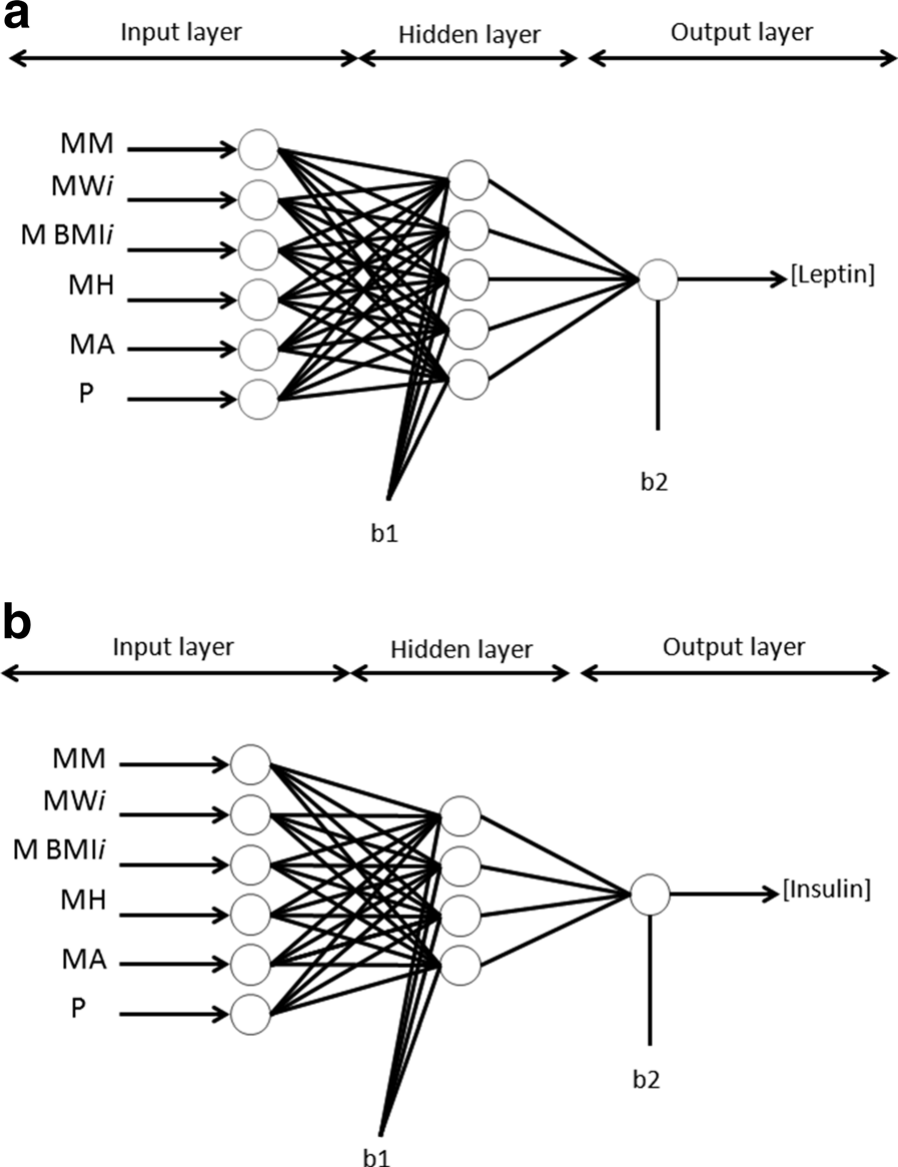
The neural network computational ANN prenatal model for UCB leptin (**a**) and insulin (**b**) concentration estimation. The proposed model involved 6 input variables, 5 neurons on hidden layer for leptin or 4 neurons on hidden layer for insulin and 1 output variable

The input variables for the ANN prenatal models (early gestation) were 6 maternal and 1 output variable for UCB leptin or insulin concentration. The best neural network architecture obtained for the prediction of UCB leptin was 6-5-1 (Fig. 5a) and for UCB insulin, 6-4-1 (Fig. 5b) (The same Equations [6–11 for leptin and 6–10 for insulin] were utilized in the model but the weights and biases for leptin and insulin simulations are reported in Additional file 1: Tables S3 and S4). The ANN models were able to predict UCB leptin and insulin levels from only anthropometric maternal parameters.

### Validation of the ANN prenatal model

**Fig. 6:**
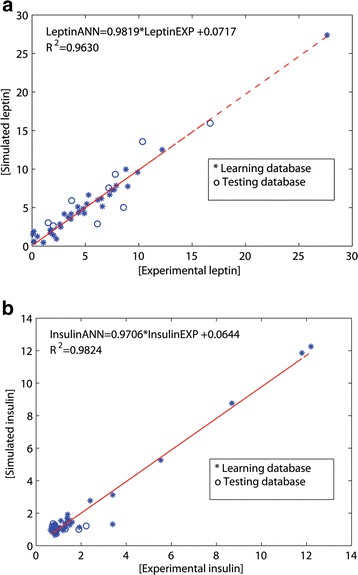
The scatter plot of prenatal experimental (open circles) vs. ANN-predicted values (dark cross) for average UCB leptin (**a**) and insulin (**b**). Experimental (leptin _EXP_ and insulin _EXP_) and simulated (leptin _ANN_ and insulin _ANN_) data. Dashed line indicates the fitted simple regression line on scattered points

Figure 6 depicts the predicted values compared to the experimental values for leptin (Fig. 6a) and insulin (Fig. 6b), showing a good capability of the model to simulate both outputs by describing the behavior of the UCB levels using only maternal anthropometric information from early gestation.

The regression coefficients were *R*^2^ > 0.963 and *R*^2^ > 0.9824 for leptin and insulin, respectively (Fig. 6). Table 5 depicts intercepts and slopes for the linear regression model of the ANN prenatal models. Therefore, these statistical results guarantee the validity of the ANN prenatal models with a confidence level of 99.8 % for both UCB leptin and insulin [39, 47].

**Table 5:** Intercept (a) and slope (b) statistical test to leptin and insulin in the ANN prenatal model

Leptin		Insulin	
a_lower_	a_upper_	a_lower_	a_upper_
_0.8508_	_1.1131_	_0.8838_	_1.0574_
b_lower_	b_upper_	b_lower_	b_upper_
_−0.8453_	_0.9886_	_−0.2069_	_0.3357_

### Sensitivity analysis of the ANN prenatal model

**Fig. 7:**
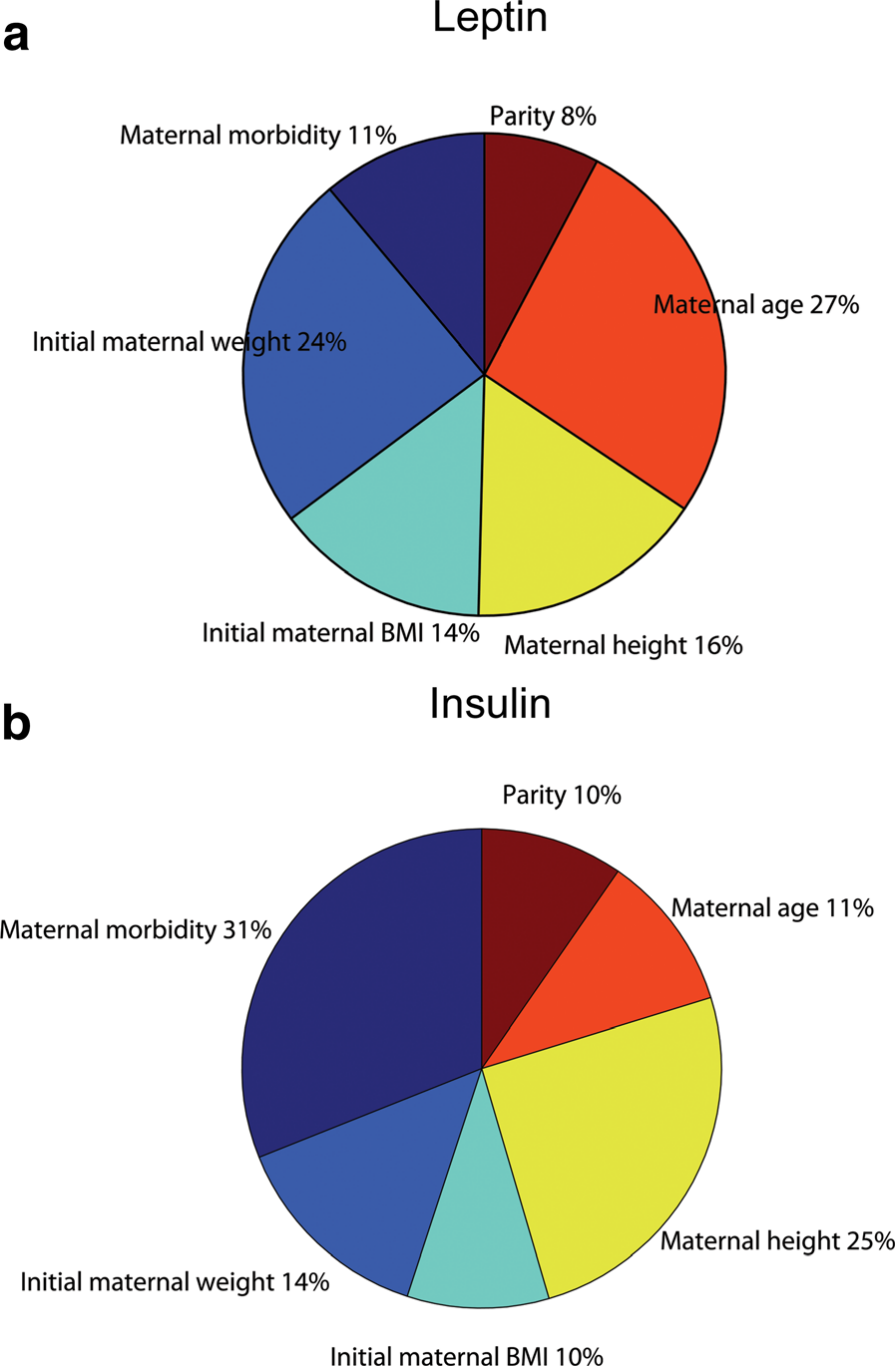
Percentage for the global sensitivity analysis of the 6 input variables in the ANN prenatal model for UCB leptin (**a**) and Insulin (**b**) values

The same process used for the ANN perinatal models allowed obtaining the relative importance of the input variables on the simulated UCB leptin (Fig. 7a) and insulin (Fig. 7b) values in the ANN prenatal models. Figure 7a shows that maternal age (27 %) and initial maternal weight (24 %) were the dominant factors for the prediction of UCB leptin in comparison with maternal morbidity (11 %) and parity (8 %), which were the less important parameters. For UCB insulin simulation (Fig. 7b), all maternal characteristics had a strong effect on insulin values but maternal morbidity (31 %) and maternal height (25 %) were the predominant parameters followed by maternal age (11 %) and parity (10 %).

## Discussion

Four ANNs models were developed to predict neonatal leptin and insulin concentrations in umbilical cord blood, based on selected experimental conditions (anthropometric and biochemical variables), from gestation and at term data (ANN perinatal models) or only from early gestation data (ANN prenatal models). The neonatal leptin and insulin parameters were successfully simulated by applying in all models, a three layered neural network with 4–5 neurons in the hidden layer, using a back-propagation algorithm that achieved a low average error rate (<3 and <4 %, for the ANN perinatal models and ANN prenatal models, respectively). The results obtained by the ANN perinatal models show a high agreement with experimental results: a good correlation (*R*^2^ > 0.97) and small error (*RMSE* > 0.0012). High level of confidence for the ANN perinatal models was confirmed with the intercept and slope statistical test (99 %). Interestingly, the ANN prenatal models, that takes into account only the early gestation information (maternal anthropometric parameters), were also able to estimate leptin and insulin values with a good correlation coefficient (*R*^2^ > 0.96 for leptin and *R*^2^ > 0.98 for insulin), a small error (*RMSE* > 0.2) and a confidence level of 99.8 %. These models consider well-known and simple to measure input parameters such as: corporal weight, length and body mass index of the mother at the beginning and/or end of pregnancy, gestational age at delivery, gender, weight, body length and head circumference of the neonate at delivery. Therefore, by means of these ANN models we could be able to obtain any unknown leptin and insulin variables based exclusively in anthropometric data.

ANN was, not only capable of establishing mathematical models estimating neonatal leptin and insulin values in umbilical cord blood from anthropometric values, but was also able to identify key maternal and neonatal variables, which had mathematically consistent biological relevance for the predicted values. According to the sensitivity analysis of the ANN perinatal models, we found that maternal BMI and neonatal birth weight were the most influential parameters for the prediction of neonatal leptin values, while maternal metabolic health was the principal factor for the simulation of neonatal insulin levels. Interestingly, the sensibility analysis of the ANN prenatal models (taking into account only early gestation maternal anthropometric values) showed maternal age and initial maternal weight had a strong impact on UCB leptin levels, whereas maternal metabolic health was the most important parameter for fetal insulin secretion.

These analyses were capable of confirming a major role of maternal BMI and birth weight for UCB leptin prediction and maternal metabolic health for insulin values. Indeed, it has been shown that UCB leptin concentration correlates with maternal BMI and neonatal birth weight by conventional observational and statistical methods [18–20, 54, 55]. In particular, maternal BMI as a key factor for UCB leptin levels is in agreement with the proposed mechanism for leptin during pregnancy. In fact, the adipose tissue, the placenta and the vascular endothelium of the mother have been demonstrated as sites for regulated leptin production *in* utero [56, 57] and during pregnancy, leptin has been shown to regulate protein synthesis, growth and immunity [58].

For UCB insulin values, maternal metabolic health (healthy, obese or obese with gestational diabetes mellitus) appears to be the critical parameter, followed by neonatal body length. Indeed, gestational diabetes mellitus exposed neonates had higher umbilical cord blood insulin levels than not exposed neonates [48, 59], which was confirmed by the ANN analysis. The fetal pancreas is the principal source of fetal insulin since maternal insulin does not cross the blood/placental barrier [60]. The fact that insulin is higher in gestational diabetes mellitus offspring may suggest dysregulation of insulin signaling at birth which is compatible with an adaptation for elevated maternal glucose levels [61]. Prediction of UCB insulin levels by neonatal body length is in agreement to insulin’s direct anabolic action. This has been proposed to be indirectly mediated via leptin, since UCB leptin levels strongly correlate with UCB insulin values [21]. Also, it has been hypothesized that fetal insulin stimulates fetal adipocyte leptin production [21]. However, UCB leptin levels did not correlate with UCB insulin levels in the offspring of obese women with gestational diabetes mellitus.

### Limitations and strengths of the ANN models

It is important to acknowledge the limitations of this study such as the particularly low sample size. However, adaptive learning algorithms like ANN were able to overcome this problem of low sample size due to the training procedure that uses only a part of the database. It is noteworthy to mention that a study from *Street and cols* used a similar size database for their ANN model in order to identify placental factors for fetal growth [36]. Another limitation of this work is the low test data size. Further simulations with an increased sample size should allow improving the ANN models.

In addition, the limits of the modeling are that in order to predict UCB leptin and insulin concentrations, morbidity, anthropometric and biochemical parameters must be placed between the ranges of the input variables (see Table 2). For example, the ANN perinatal model will accurately simulate UCB leptin when applied to mothers with an initial weight (MW_*i*_) comprised between 49–96 kg and an initial BMI (MBMI_*i*_) of 22–40 kg/m^2^.

One of the strengths of this ANN approach is that the elapsed time to calculate both neonatal parameters is short which can be applied on-line and that it represents the dynamic interactions of complex relationships. These characteristics suggest a possible translational utility of these ANN models.

Insulin and leptin cord blood levels have been used as predictor of postnatal growth and weight gain in infancy. So, the establishment of ANN prenatal models that predicts these values from maternal anthropometric variables during gestation, without the need for cord blood samples, could be helpful to prognosticate infant growth and permit the possibility of conducting interventions. For example, it is tempting to speculate that infants from obese mothers that had the highest cord leptin values will probably have the worst of all weight gain but the highest BMI in infancy, since higher cord blood leptin and gestational diabetes are related to a slower weight gain and an increased BMI at 2–3 years [23, 24]. The use of these ANN models could make easy to follow growth and perhaps estimate the risk of obesity and diabetes in these children.

## Conclusions

Low error percentage and short computing time makes this ANN models attractive to be applied for the prediction of UCB leptin and insulin values from maternal and neonatal anthropometric data, and possibly the on-line estimation during pregnancy, birth and infancy. In particular, the prediction of these hormone values in UCB may be of great interest to prognosticate infant growth and permit the possibility of conducting interventions before incurring costly and time-consuming events, such as neonatal morbidity. Moreover, the fact that the ANN prenatal model, based merely on early gestation anthropometric maternal information, was able to confidently simulate UCB leptin and insulin levels, make these particular models an interesting application for following the impact of maternal anthropometrics and metabolic health on these hormone UCB values and to predict leptin and insulin values at birth from early gestation.

## Abbreviations

ANN, artificial neural network; BP, back-propagation algorithm; b, biases; BMI, body mass index; _EXP,_ experimentally measured; MBMf, final maternal BMI; MWf, final maternal weight; GE, gestational age at delivery; TANSIG, hyperbolic tangential transfer function; MBMi, initial maternal BMI; MWi, initial maternal weight; LM, Levenberg-Marquardt algorithm; LOGSIG, log-sigmoid transfer function; MA, maternal age; MH, maternal height; MM, maternal morbidity; MLP, multiple-layer perceptron; NW, neonatal birth weight; NBMI, neonatal BMI; NH, neonatal body length; NG, neonatal gender; NHC, neonatal head circumference; P, parity; _ANN_, predicted; RMSE, root mean square error; UCB, umbilical cord blood; W, weights.

## Additional file

Additional file 1:
**Table S1. **Weights and biases for the ANN perinatal model to predict UCB leptin concentration. Weights and biases for the network architecture performance of 12-5-1 for leptin ANN perinatal model. Table S2. Weights and biases for the ANN perinatal model to predict UCB insulin concentration. Weights and biases for the network architecture performance of 12-5-1 for insulin ANN perinatal model. Table S3. Weights and biases for the ANN prenatal model to predict UCB leptin concentration. Weights and biases for the network architecture performance of 6-5-1 for leptin ANN prenatal model. Table S4. Weights and biases for the ANN prenatal model to predict UCB insulin concentration. Weights and biases for the network architecture performance of 6-4-1 for insulin ANN prenatal model. (DOCX 60 kb)
