# Supplementary material for: Estimation of umbilical cord blood leptin and insulin based on anthropometric data by means of artificial neural network approach: identifying key maternal and neonatal factors
Source: BMC Pregnancy Childbirth. 2016 Jul 21;16:179. doi: 10.1186/s12884-016-0967-z (PMC4955136; doi:10.1186/s12884-016-0967-z)
Supplement: Additional file 1: — Table S1. Weights and biases for the ANN perinatal model to predict UCB leptin concentration. Weights and biases for the network architecture performance of 12-5-1 for leptin ANN perinatal model. Table S2. Weights and biases for the ANN perinatal model to predict UCB insulin concentration. Weights and biases for the network architecture performance of 12-5-1 for insulin ANN perinatal model. Table S3. Weights and biases for the ANN prenatal model to predict UCB leptin concentration. Weights and biases for the network architecture performance of 6-5-1 for leptin ANN prenatal model. Table S4. Weights and biases for the ANN prenatal model to predict UCB insulin concentration. Weights and biases for the network architecture performance of 6-4-1 for insulin ANN prenatal model. (DOCX 60 kb) [file 12884_2016_967_MOESM1_ESM.docx]

**SUPLEMENTARY INFORMATION**

**METHODS**

***ANN model***

In the hidden and output layer, each neuron (*n*) has weights (*Wi* and *Wo)* and biases (b1 and b2), see equations 1 and 2:

*n_1_* = *WiIn*_1_ + *WiIn*_2_ +…….+*WiIn*_k_ + *b_1_* (1)

The value of each neuron is the argument of the transfer functions (*f* and *g*):

*Output = g(Wo × f (Wi × In + b1) + b2)* (2)

Where *f* is a hyperbolic tangent transfer function (TANSIG) and *g* is a linear transfer function (PURELIN). As a result of Equation [2]:

$output_{l}=\sum_{s} \left\{Wo_{(l,s)}\cdot\left[\frac{2}{1+e^{-2\cdot\left(\sum_{k} Wi_{(s,k)}\cdot In_{k}+b1_{(s,1)}\right)}}-1\right]\right\}+b2_{(l,1)}$ (3)

***ANN Learning***

In this study, to change the weights and biases, we applied the Levenberg-Marquardt (LM), which uses the adaptation as follows:

$\Delta w=\left( J^{T}J+\mu I \right)^{-1}J^{T}e$ . Where *J* is the Jacobian matrix (first derivative), *e* is a vector of network errors, *μ* is the combination coefficient with a value of 0.001 and *I* is the identity matrix.

The Root Mean Square Error $\left(RMSE\right)$ [Eq.4] was applied as the error function which describes the performance of the network according to the following equation:

$RMSE=\sqrt{\frac{\left(\sum_{q=1}^{Q} \left(y_{q,\exp}-y_{q,ANNsim}\right)^{2}\right)}{Q}}$ (4)

Where:

Q is the number of data points (n=49),

$y_{q,\exp}$ is the experimental data,

$y_{q,ANNsim}$ is the network prediction.

**RESULTS**

***General ANN models***

The proposed ANNs models follow Equation [5]:

$Insulin\left(or\right)Leptin=\sum_{s=1}^{S} \left[Wo_{(1,s)}\left(\frac{2}{1+\exp\left(-2\left(\sum_{k=1}^{K} \left(Wi_{(s,k)}In_{(k)}\right)+b1_{(s)}\right)\right)}-1\right)\right]+b2_{(1)}$ (5)

The following Equation [5] gives neonatal leptin or insulin concentrations with weights and biases from Table S1 (Leptin ANN perinatal model, 12-5-1), Table S2 (Insulin ANN perinatal model, 12-5-1), Table S3 (Leptin ANN prenatal model, 6-5-1) and Table S4 (Insulin ANN prenatal model, 6-4-1).

**Table S1. Weights and biases for the ANN perinatal model to predict UCB leptin concentration.**

| 5 neurons on hidden layer (k=12 and l=1) | | | | | | | | | | | | |
| --- | --- | --- | --- | --- | --- | --- | --- | --- | --- | --- | --- | --- |
|  | Wi_{s,1}_ | Wi_{s,2}_ | Wi_{s,3}_ | Wi_{s,4}_ | Wi_{s,5}_ | Wi_{s,6}_ | Wi_{s,7}_ | Wi_{s,8}_ | Wi_{s,9}_ | Wi_{s,10}_ | Wi_{s,11}_ | Wi_{s,12}_ |
| Wi_{s,k}_ | -0.0414 | 0.3297 | 0.1394 | 0.3135 | -2.6251 | 3.1275 | -1.3825 | -3.0800 | 4.0875 | 1.1547 | -0.7645 | 0.8333 |
|  | -0.4506 | 4.3293 | -4.9120 | -3.9748 | -1.2038 | -4.3650 | -0.1543 | -2.4516 | 0.7993 | -1.0971 | -2.0351 | 0.8604 |
|  | -2.7030 | -0.1906 | -0.8187 | -2.6255 | -1.9781 | -3.2550 | 1.2236 | -0.8837 | -3.9756 | -1.6992 | 1.4099 | 0.0171 |
|  | 0.8193 | 0.4923 | -2.7669 | -3.5464 | 4.1888 | -3.7055 | 1.3168 | 3.4650 | -0.9034 | 2.1138 | 0.3218 | -0.2031 |
|  | -0.7120 | -0.5637 | -4.8627 | -1.4219 | -5.9596 | 0.6566 | 0.3778 | -3.8291 | -1.9944 | 0.3594 | -0.1092 | 0.6784 |
| Wo_{l,s}_ | Wo_{1,1}_  5.8730 | Wo_{1,2}_  -9.1495 | Wo_{1,3}_  -13.017 | Wo_{1,4}_  6.4128 | Wo_{1,5}_  7.4967 |  |  |  |  |  |  |  |
| b1_{s,1}}_ | b1_{5,1}_  1.2703  5.0607  11.1918  1.6385  7.8402 |  |  |  |  |  |  |  |  |  |  |  |
| b2_{l,s}_ | b2_{1,1}_  4.4985 |  |  |  |  |  |  |  |  |  |  |  |

**Table S2. Weights and biases for the ANN perinatal model to predict UCB insulin concentration.**

| 5 neurons on hidden layer (k=12 and l=1) | | | | | | | | | | | | |
| --- | --- | --- | --- | --- | --- | --- | --- | --- | --- | --- | --- | --- |
|  | Wi_{s,1}_ | Wi_{s,2}_ | Wi_{s,3}_ | Wi_{s,4}_ | Wi_{s,5}_ | Wi_{s,6}_ | Wi_{s,7}_ | Wi_{s,8}_ | Wi_{s,9}_ | Wi_{s,10}_ | Wi_{s,11}_ | Wi_{s,12}_ |
| Wi_{s,k}_ | 3.4392 | -4.1536 | -2.3705 | 2.4662 | -0.4430 | -0.7171 | 4.7391 | 1.8624 | 2.0888 | 5.5695 | 0.2059 | -1.6593 |
|  | -7.2271 | -1.7251 | 2.0490 | -0.5846 | -1.4554 | -3.7848 | 1.5945 | 2.2606 | 1.0722 | 5.5406 | 0.3909 | -1.9750 |
|  | 5.5298 | -5.1711 | 4.1226 | -7.4891 | 3.2778 | -8.7547 | 3.0309 | 0.4369 | -0.6961 | 3.6414 | -1.7849 | 1.3245 |
|  | -9.8211 | 1.0311 | 1.3927 | 4.3929 | -2.3555 | 1.5897 | 1.4138 | 1.2179 | 3.2259 | 2.3797 | 4.0983 | -1.6136 |
|  | -0.9400 | -1.9047 | -0.6777 | 0.8357 | 3.3487 | 0.1366 | -2.1174 | -1.6266 | 0.1438 | 0.2745 | -7.3589 | -2.4671 |
| Wo_{l,s}_ | Wo_{1,1}_  6.8563 | Wo_{1,2}_  7.1133 | Wo_{1,3}_  -7.3257 | Wo_{1,4}_  -7.2597 | Wo_{1,5}_  -2.7748 |  |  |  |  |  |  |  |
| b1_{s,1}}_ | b1_{5,1}_  -2.0747  2.4494  3.2189  -2.4255  -1.4636 |  |  |  |  |  |  |  |  |  |  |  |
| b2_{l,s}_ | b2_{1,1}_  -1.3633 |  |  |  |  |  |  |  |  |  |  |  |

**Table S3. Weights and biases for the ANN prenatal model to predict UCB leptin concentration.**

| 5 neurons on hidden layer (k=6 and l=1) | | | | | | |
| --- | --- | --- | --- | --- | --- | --- |
|  | Wi_{s,1}_ | Wi_{s,2}_ | Wi_{s,3}_ | Wi_{s,4}_ | Wi_{s,5}_ | Wi_{s,6}_ |
| Wi_{s,k}_ | 7.6519 | -6.8235 | 1.1395 | 0.3203 | -3.7955 | -5.8509 |
|  | -7.9849 | 7.0292 | 0.8047 | -4.1815 | 1,9427 | -1.9939 |
|  | 2.0117 | -10.985 | 8.5582 | 9.2839 | 11.5297 | -1.4040 |
|  | 1.876 | 4.4392 | -11.850 | -14.153 | 17.2573 | -4.1181 |
|  | -0.0395 | 10.9663 | -7.7886 | -5.7010 | -17.610 | 2.7110 |
| Wo_{l,s}_ | Wo_{1,1}_  -6.9031 | Wo_{1,2}_  -11.934 | Wo_{1,3}_  -16.637 | Wo_{1,4}_  -6.4600 | Wo_{1,5}_  -21.792 |  |
| b1_{s,1}}_ | b1_{5,1}_  2.4775  6.1135  -8.1117  2.0695  7.6746 |  |  |  |  |  |
| b2_{l,s}_ | b2_{1,1}_  10.4735 |  |  |  |  |  |

**Table S4. Weights and biases for the ANN prenatal model to predict UCB insulin concentration.**

| 4 neurons on hidden layer (k=6 and l=1) | | | | | | |
| --- | --- | --- | --- | --- | --- | --- |
|  | Wi_{s,1}_ | Wi_{s,2}_ | Wi_{s,3}_ | Wi_{s,4}_ | Wi_{s,5}_ | Wi_{s,6}_ |
| Wi_{s,k}_ | 12.0266 | -2.5600 | -1.5536 | 3.1853 | -5.4324 | 4.6368 |
|  | -9.8459 | 7.8881 | -0.7121 | -21.357 | -7.5965 | 1.3454 |
|  | 8.2764 | -6.4111 | -12.756 | -17.113 | 0.2120 | -7.0533 |
|  | 5.8404 | -2.1400 | 0.4710 | 0.2398 | 0.4904 | -0.3226 |
| Wo_{l,s}_ | Wo_{1,1}_  -11.199 | Wo_{1,2}_  -11.143 | Wo_{1,3}_  10.9673 | Wo_{1,4}_  5.7906 |  |  |
| b1_{s,1}}_ | b1_{5,1}_  -7.1804  16.7212  6.5591  2.3989 |  |  |  |  |  |
| b2_{l,s}_ | b2_{1,1}_  6.4173 |  |  |  |  |  |

As an example for developing simulated leptin by ANN perinatal model, we describe the following (considering the input variables as well as Wo, Wi, b1 and b2 of Table S1):

*V_1_=maternal morbidity; V_2_=gestational age at delivery; V_3_=initial maternal weight; V_4_=final maternal weight; V_5_=initial maternal BMI; V_6_=final maternal BMI; V_7_=maternal height; V_8_=neonatal gender; V_9_=birth weight; V_10_=birth body length; V_11_=birth head circumference; V_12_=neonatal BMI*

$Leptin = 2 \left[\frac{Wo_{(1,1)}}{1+e^{X1}}+\frac{Wo_{(1,2)}}{1+e^{X2}}+\frac{Wo_{(1,3)}}{1+e^{X3}}+\frac{Wo_{(1,4)}}{1+e^{X4}}+\frac{Wo_{(1,5)}}{1+e^{X5}}\right] -\left(Wo_{(1,1)}+Wo_{(1,2)}+Wo_{(1,3)}+Wo_{(1,4)}+Wo_{(1,5)}\right)+b2_{(1)}$ (6)

where:

$X1 =-2 \left(\begin{matrix} W_{i(1,1)}V_{1}+W_{i(1,2)}V_{2}+W_{i(1,3)}V_{3}+W_{i(1,4)}V_{4}+W_{i(1,5)}V_{5}+W_{i(1,6)}V_{6}+W_{i(1,7)}V_{7}... \\ +W_{i(1,8)}V_{8}+W_{i(1,9)}V_{9}+W_{i(1,10)}V_{10}+W_{i(1,11)}V_{11}+W_{i(1,12)}V_{12}+b1_{(1)} \end{matrix}\right)$ (7)

$X2 =-2 \left(\begin{matrix} W_{i(2,1)}V_{1}+W_{i(2,2)}V_{2}+W_{i(2,3)}V_{3}+W_{i(2,4)}V_{4}+W_{i(2,5)}V_{5}+W_{i(2,6)}V_{6}+W_{i(2,7)}V_{7}... \\ +W_{i(2,8)}V_{8}+W_{i(2,9)}V_{9}+W_{i(2,10)}V_{10}+W_{i(2,11)}V_{11}+W_{i(2,12)}V_{12}+b1_{(2)} \end{matrix}\right)$ (8)

$X3 =-2 \left(\begin{matrix} W_{i(3,1)}V_{1}+W_{i(3,2)}V_{2}+W_{i(3,3)}V_{3} +W_{i(3,4)}V_{4}+W_{i(3,5)}V_{5}+W_{i(3,6)}V_{6}+W_{i(3,7)}V_{7}... \\ +W_{i(3,8)}V_{8}+W_{i(3,9)}V_{9}+W_{i(3,10)}V_{10}+W_{i(3,11)}V_{11}+W_{i(3,12)}V_{12}+b1_{(3)} \end{matrix}\right)$ (9)

$X4=-2 \left(\begin{matrix} W_{i(4,1)}V_{1}+W_{i(4,2)}V_{2}+W_{i(4,3)}V_{3} +W_{i(4,4)}V_{4}+W_{i(4,5)}V_{5}+W_{i(4,6)}V_{6}+W_{i(4,7)}V_{7}... \\ +W_{i(4,8)}V_{8}+W_{i(4,9)}V_{9}+W_{i(4,10)}V_{10}+W_{i(4,11)}V_{11}+W_{i(4,12)}V_{12}+b1_{(4)} \end{matrix}\right)$ (10)

$X5 =-2 \left(\begin{matrix} W_{i(5,1)}V_{1}+W_{i(5,2)}V_{2}+W_{i(5,3)}V_{3} +W_{i(5,4)}V_{4}+W_{i(5,5)}V_{5}+W_{i(5,6)}V_{6}+W_{i(5,7)}V_{7}... \\ +W_{i(5,8)}V_{8}+W_{i(5,9)}V_{9}+W_{i(5,10)}V_{10}+W_{i(5,11)}V_{11}+W_{i(5,12)}V_{12}+b1_{(5)} \end{matrix}\right)$ (11)

Similarly, the ANN prenatal model used Equations [6-11 for leptin and 6-10 for insulin] and considered the weights and biases of Table S3 and S4, respectively.

***General Sensitivity analysis***

The author [1-2] proposed an equation based on the partitioning of connection weights:

$I_{j}= \frac{\sum_{m=1}^{Nh} \left(\left(\frac{\left|W_{jm}^{ih}\right|}{\sum_{k=1}^{Ni} \left|W_{km}^{ih}\right|}\right)\times\left|W_{mn}^{ho}\right|\right)}{\sum_{k=1}^{Ni} \left\{\sum_{m=1}^{Nh} \left(\frac{\left|W_{km}^{ih}\right|}{\sum_{k=1}^{Ni} \left|W_{km}^{ih}\right|}\right)\times\left|W_{mn}^{ho}\right|\right\}}$ (12)

Where:

$I_{j}$is the relative importance of the $jth$ input variable on the output variable,

$N_{i}$is the number of input neurons,

$N_{h}$ is the number of hidden neurons,

$W$ is the connection weight,

And the superscripts $'i','h'$ and $'o'$refer to input, hidden and output neurons, respectively.
